# Supplementary material for: New and Preliminary Evidence on Altered Oral and Gut Microbiota in Individuals with Autism Spectrum Disorder (ASD): Implications for ASD Diagnosis and Subtyping Based on Microbial Biomarkers
Source: Nutrients. 2019 Sep 6;11(9):2128. doi: 10.3390/nu11092128 (PMC6770733; doi:10.3390/nu11092128)

Differential abundance of gut microbiome in ASD and control subjects at phylum level

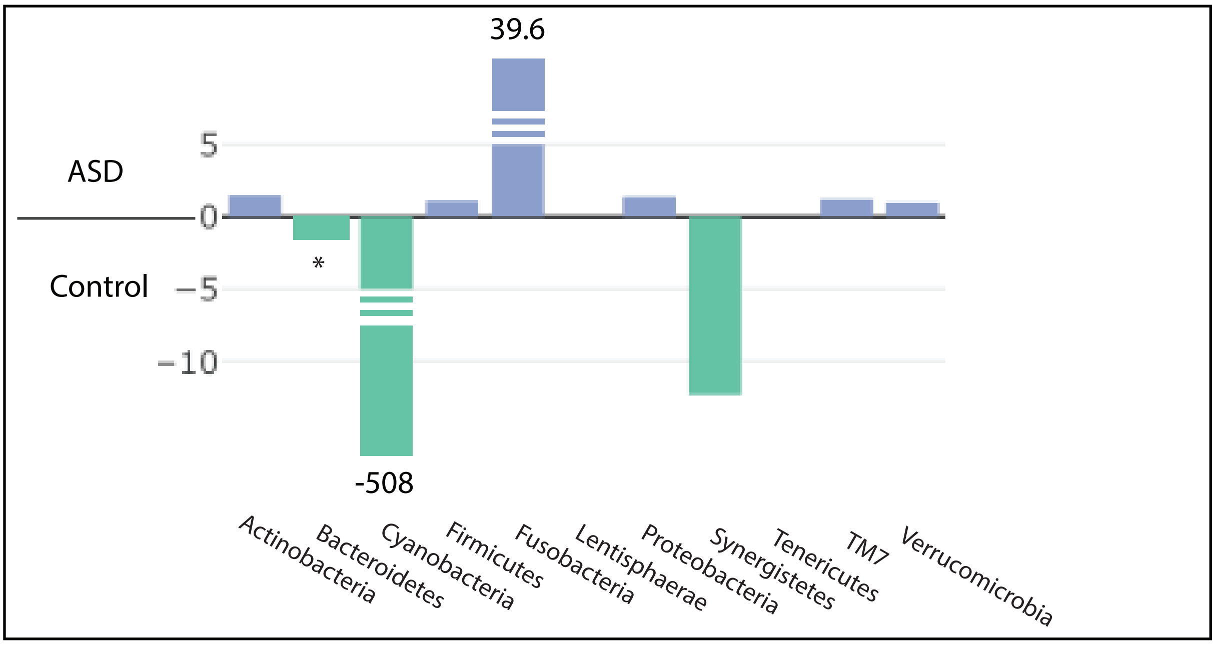

Differential abundance of salivary microbiome in ASD and control subjects at phylum level

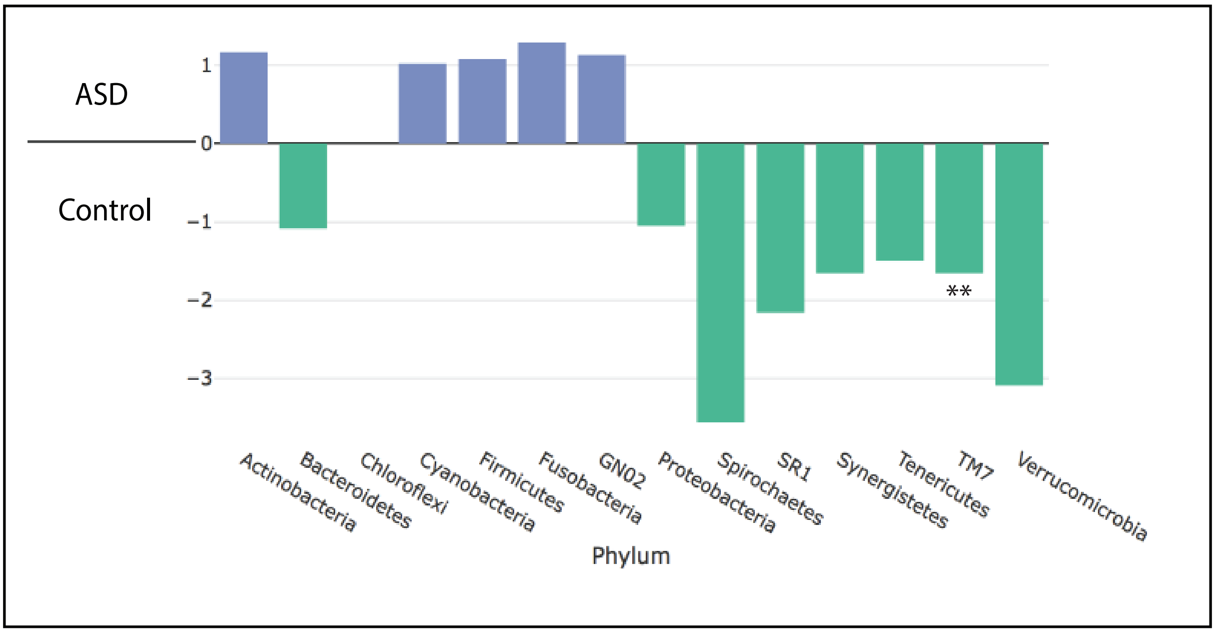

Supplement: Supplementary file 1 [file nutrients-11-02128-s001.zip › Supplementary/supplementary figures PDF/Additional file 4_SuppFig4.pdf]
