# Supplementary material for: New and Preliminary Evidence on Altered Oral and Gut Microbiota in Individuals with Autism Spectrum Disorder (ASD): Implications for ASD Diagnosis and Subtyping Based on Microbial Biomarkers
Source: Nutrients. 2019 Sep 6;11(9):2128. doi: 10.3390/nu11092128 (PMC6770733; doi:10.3390/nu11092128)
Supplement: Supplementary file 1 [file nutrients-11-02128-s001.zip › Supplementary/supplementary figures PDF/Additional file 3_SuppFig3.pdf]

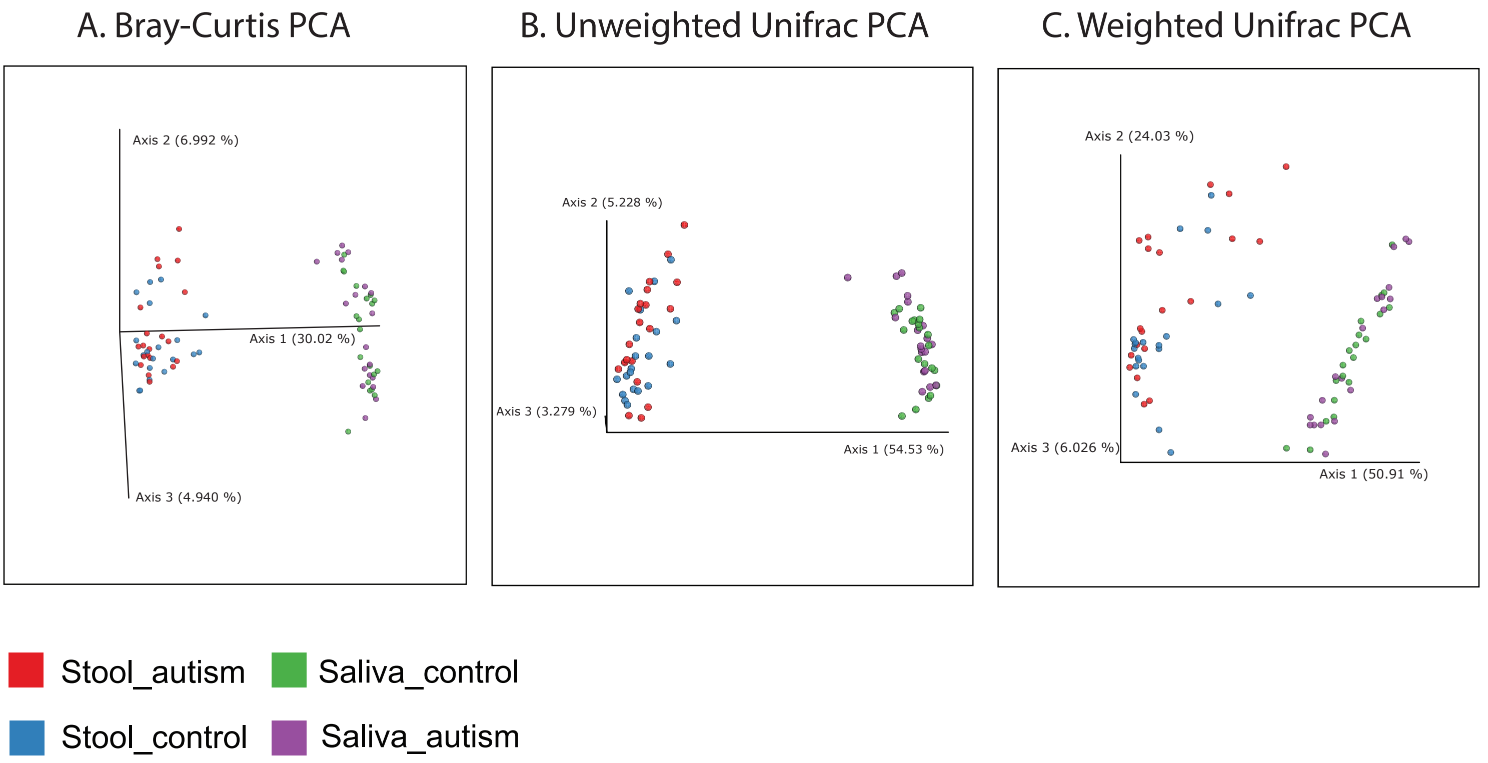
A. Bray-Curtis PCA
Axis 2 (6.992 %)
Axis 1 (30.02 %)
Axis 3 (4.940 %)
B. Unweighted Unifrac PCA
Axis 2 (5.228 %)
Axis 3 (3.279 %)
Axis 1 (54.53 %)
C. Weighted Unifrac PCA
Axis 2 (24.03 %)
Axis 3 (6.026 %)
Axis 1 (50.91 %)
Stool_autism
Saliva_control
Stool_control
Saliva_autism
