# Supplementary material for: New and Preliminary Evidence on Altered Oral and Gut Microbiota in Individuals with Autism Spectrum Disorder (ASD): Implications for ASD Diagnosis and Subtyping Based on Microbial Biomarkers
Source: Nutrients. 2019 Sep 6;11(9):2128. doi: 10.3390/nu11092128 (PMC6770733; doi:10.3390/nu11092128)

A. Gut and oral microbiome genus level SparCC co-occurrence network, Controls (Corr>0.3)

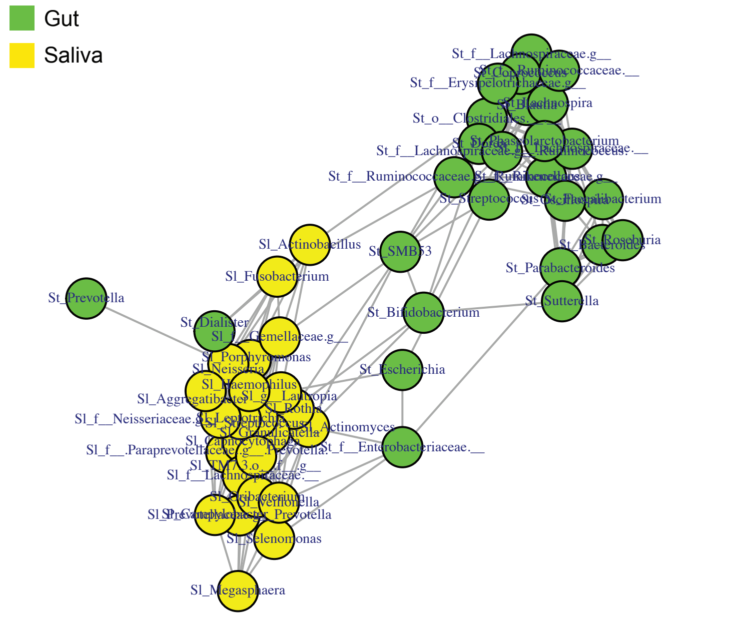

B. Gut and oral microbiome genus level SparCC co-occurrence network, ASD (Corr>0.3)

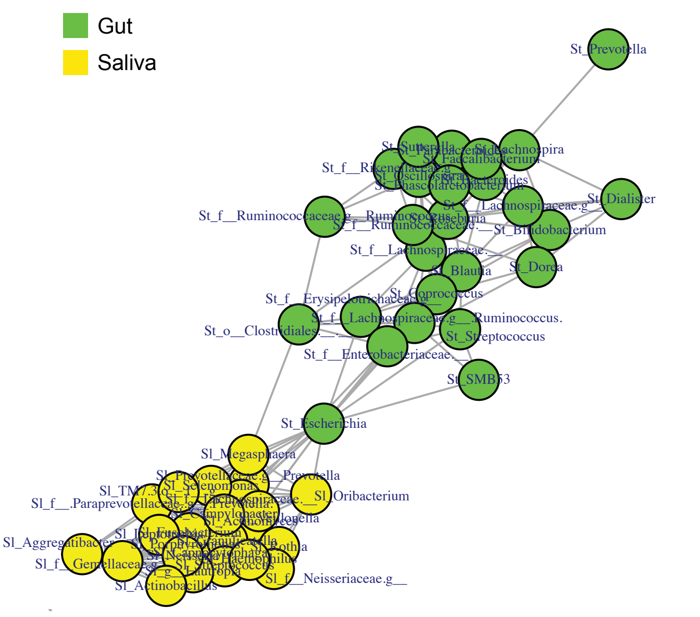

C. Gut and oral microbiome genus level SparCC co-occurrence network, Controls (Corr>0.7)

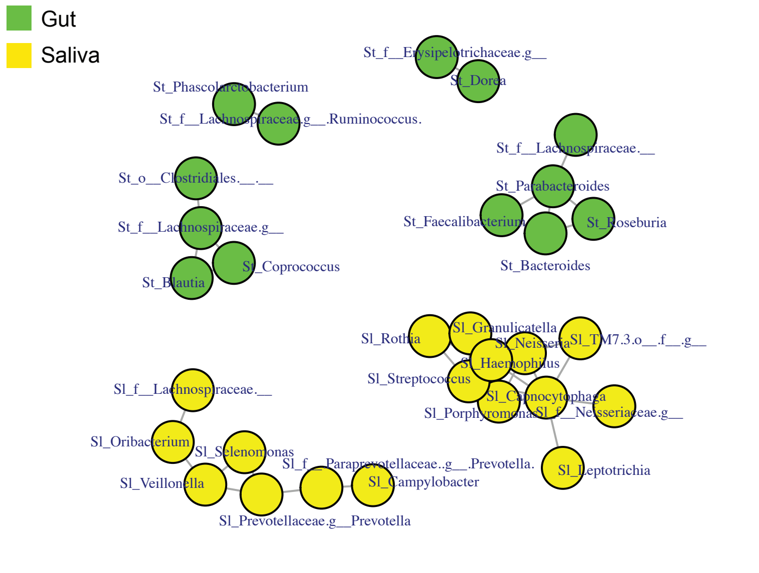

D. Gut and oral microbiome genus level SparCC co-occurrence network, ASD (Corr>0.7)

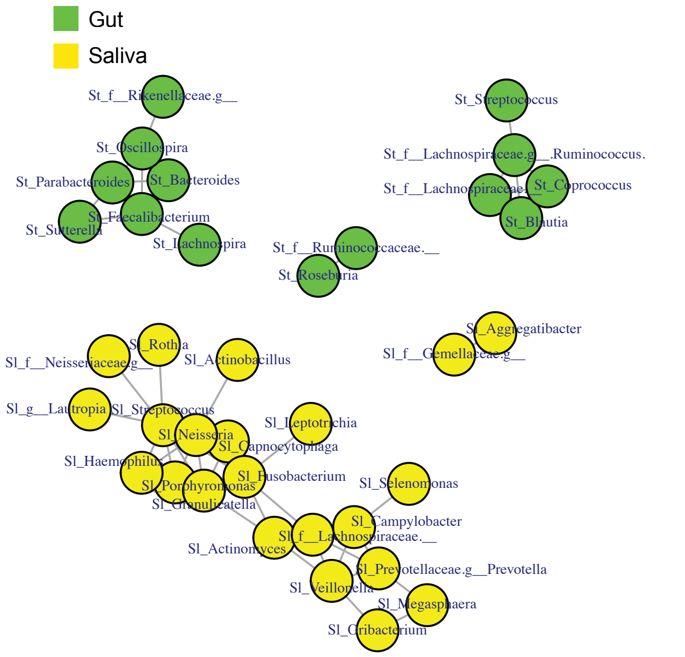

Supplement: Supplementary file 1 [file nutrients-11-02128-s001.zip › Supplementary/supplementary figures PDF/Additional file 7_SuppFig7.pdf]
