# Supplementary figures and images for: New and Preliminary Evidence on Altered Oral and Gut Microbiota in Individuals with Autism Spectrum Disorder (ASD): Implications for ASD Diagnosis and Subtyping Based on Microbial Biomarkers
Source: Nutrients. 2019 Sep 6;11(9):2128. doi: 10.3390/nu11092128 (PMC6770733; doi:10.3390/nu11092128)

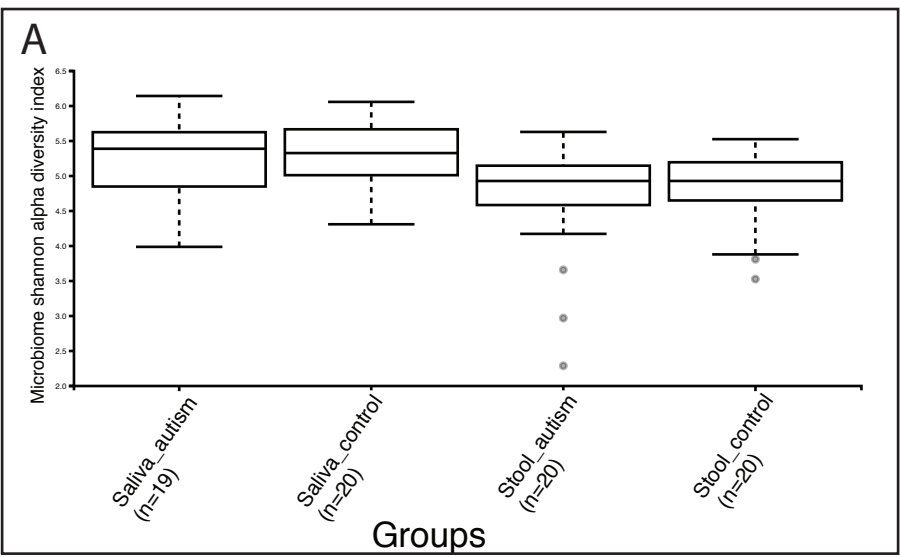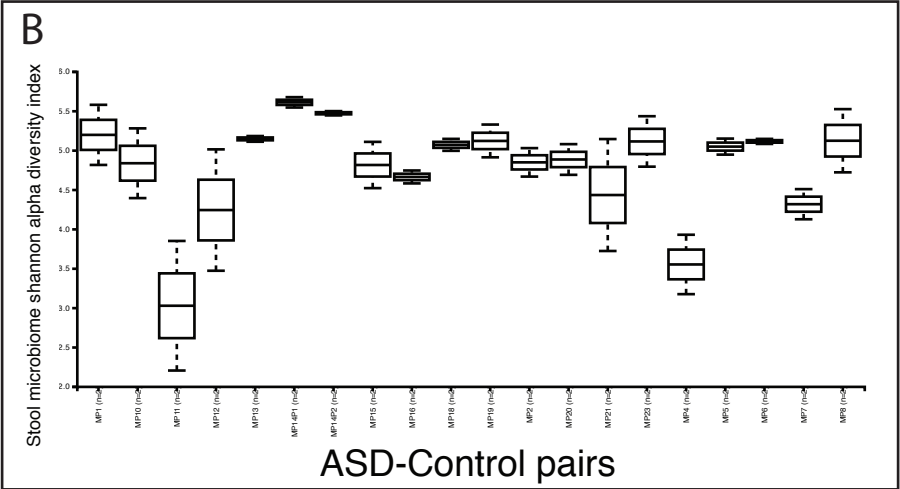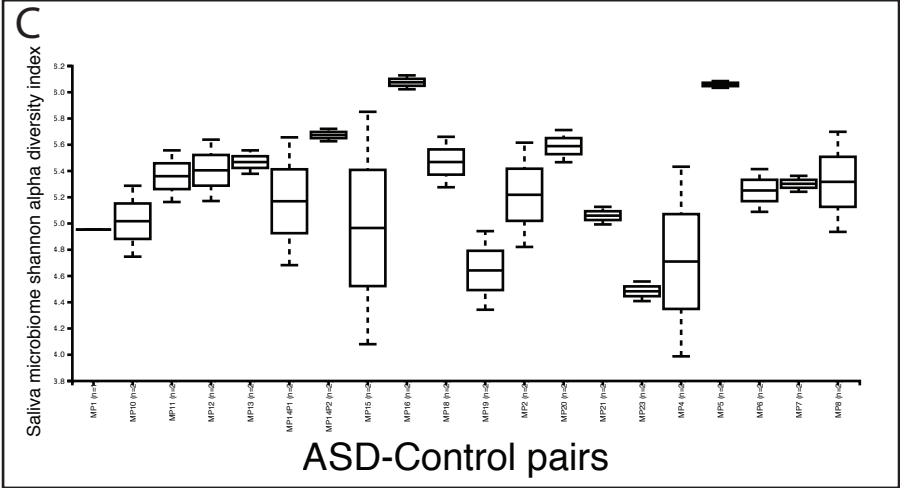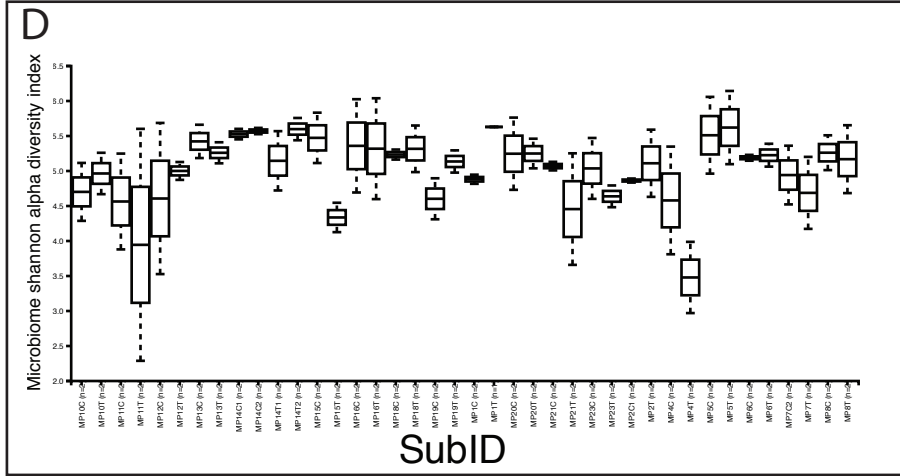

Supplement: Supplementary file 1 [file nutrients-11-02128-s001.zip › Supplementary/supplementary figures PDF/Additional file 2_SuppFig2.pdf]

A. Control gut vs. saliva ANCOM

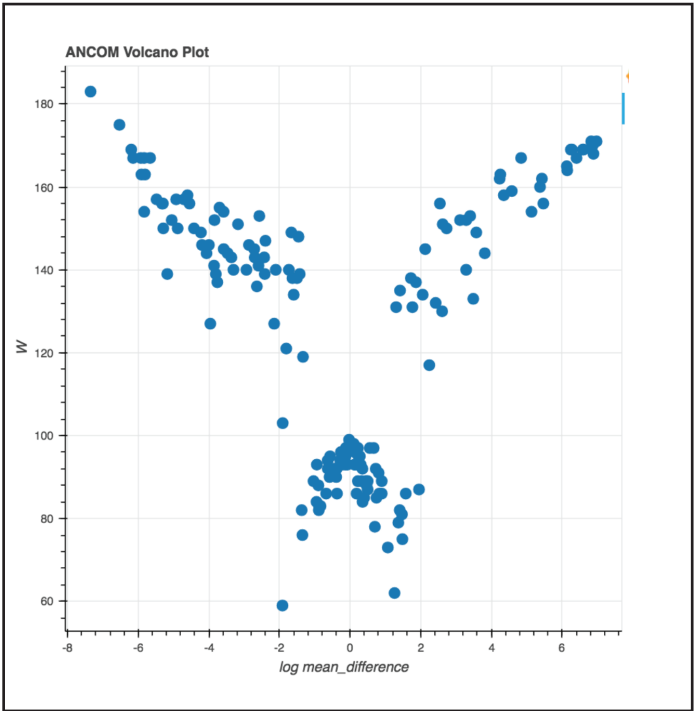

B. ASD gut vs. saliva ANCOM

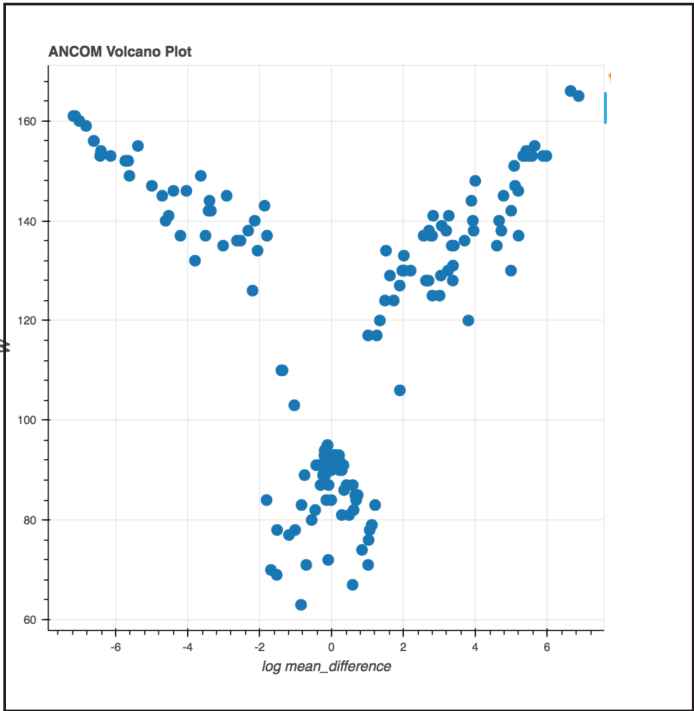

Supplement: Supplementary file 1 [file nutrients-11-02128-s001.zip › Supplementary/supplementary figures PDF/Additional file 5_SuppFig5.pdf]

Saliva microbiome  
OTU-level relative abundance

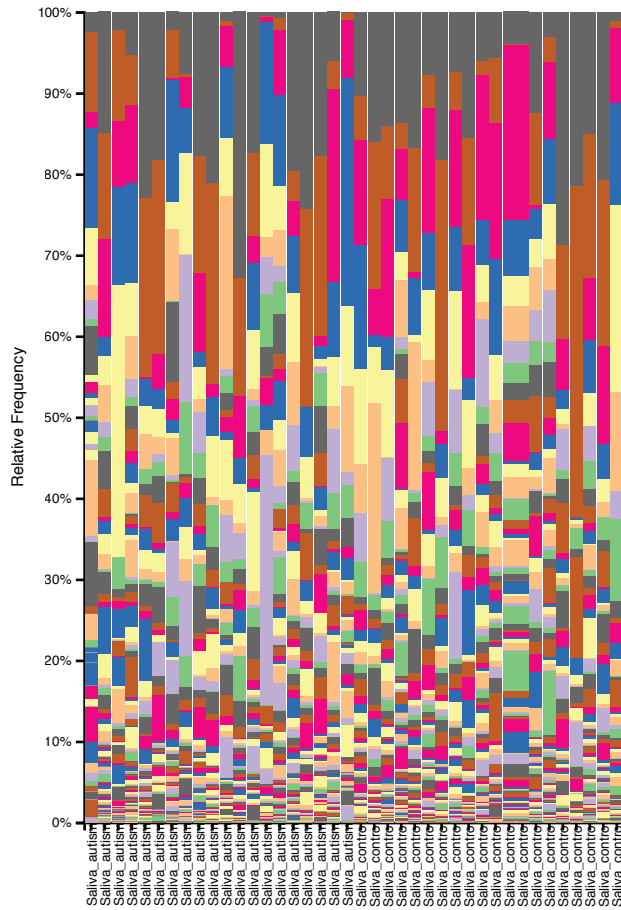

Gut microbiome  
OTU-level relative abundance

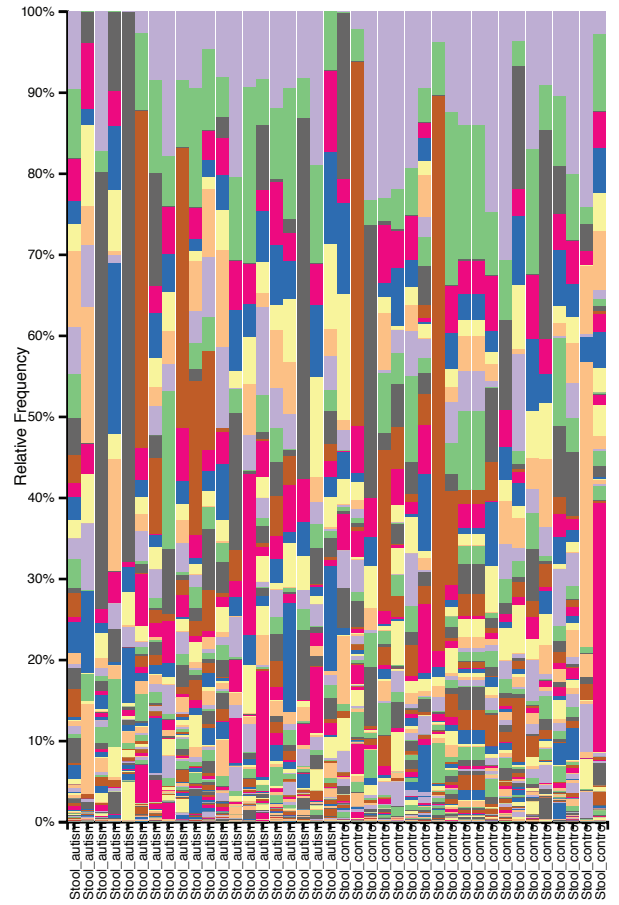

Supplement: Supplementary file 1 [file nutrients-11-02128-s001.zip › Supplementary/supplementary figures PDF/Additional file 1_SuppFig1.pdf]

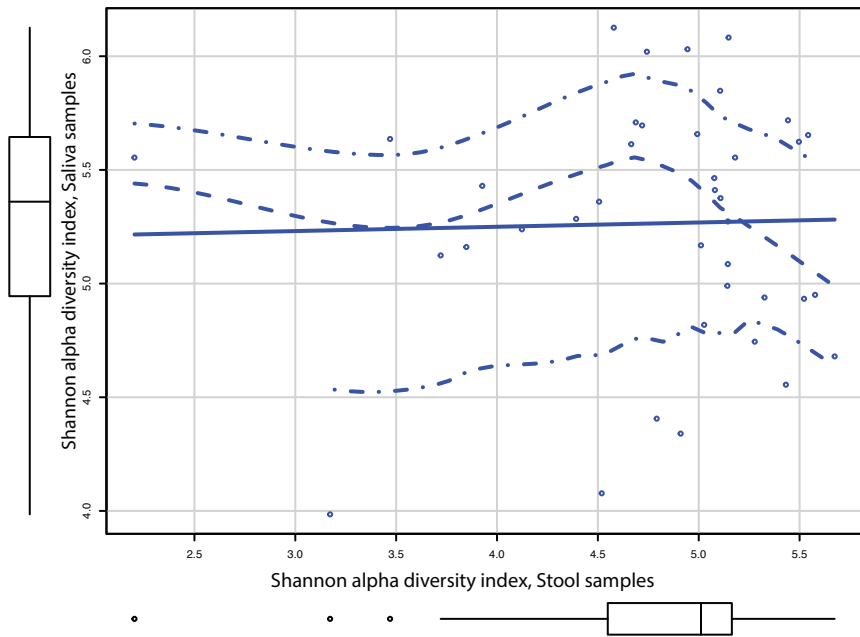

Supplement: Supplementary file 1 [file nutrients-11-02128-s001.zip › Supplementary/supplementary figures PDF/Additional file 8_SuppFig8.pdf]
